# Comprehensive identification of *Vibrio vulnificus* genes required for growth in human serum

**DOI:** 10.1080/21505594.2018.1455464

**Published:** 2018-06-21

**Authors:** M. Carda-Diéguez, F. X. Silva-Hernández, T. P. Hubbard, M. C. Chao, M. K. Waldor, C. Amaro

**Affiliations:** a Department of Microbiology and Ecology, University of Valencia. Dr. Moliner 50, Burjassot, Spain; b ERI BIOTECMED, Universitat de València. Dr Moliner 50, Burjassot, Spain; c Division of Infectious Disease, Brigham and Women's Hospital, Boston, Massachusetts, United States of America; d Howard Hughes Medical Institute, Boston, Massachusetts, United States of America; e Department of Microbiology and Immunobiology, Harvard Medical School, Boston, Massachusetts, United States of America

**Keywords:** capsule, resistance to human complement, septicaemia, transposon insertion sequencing (TIS), *Vibrio vulnificus*

## Abstract

*Vibrio vulnificus* can be a highly invasive pathogen capable of spreading from an infection site to the bloodstream, causing sepsis and death. To survive and proliferate in blood, the pathogen requires mechanisms to overcome the innate immune defenses and metabolic limitations of this host niche. We created a high-density transposon mutant library in YJ016, a strain representative of the most virulent *V. vulnificus* lineage (or phylogroup) and used transposon insertion sequencing (TIS) screens to identify loci that enable the pathogen to survive and proliferate in human serum. Initially, genes underrepresented for insertions were used to estimate the *V. vulnificus* essential gene set; comparisons of these genes with similar TIS-based classification of underrepresented genes in other vibrios enabled the compilation of a common *Vibrio* essential gene set. Analysis of the relative abundance of insertion mutants in the library after exposure to serum suggested that genes involved in capsule biogenesis are critical for YJ016 complement resistance. Notably, homologues of two genes required for YJ016 serum-resistance and capsule biogenesis were not previously linked to capsule biogenesis and are largely absent from other *V. vulnificus* strains. The relative abundance of mutants after exposure to heat inactivated serum was compared with the findings from the serum screen. These comparisons suggest that in both conditions the pathogen relies on its Na^+^ transporting NADH-ubiquinone reductase (NQR) complex and type II secretion system to survive/proliferate within the metabolic constraints of serum. Collectively, our findings reveal the potency of comparative TIS screens to provide knowledge of how a pathogen overcomes the diverse limitations to growth imposed by serum.

## Introduction

*Vibrio vulnificus* is an aquatic, gram-negative bacterium capable of causing a range of pathologies upon infecting fish or human hosts [1,2]. *V. vulnificus* infection of fish occurs principally in aquaculture, when outbreaks of hemorrhagic septicemia are perpetuated by *V. vulnificus* transmission through water or direct contact among animals. In humans, two distinct types of disease –severe skin lesions and septicemia– commonly result from *V. vulnificus* infection. Skin lesions develop following exposure of wounds to seawater or marine animals colonized by *V. vulnificus*, whereas septicemia results from ingestion of seafood contaminated by the pathogen [1]. Wound infections can also result in septicemia, particularly in immunocompromised individuals and those with high blood iron levels associated with chronic liver diseases, who exhibit 80-fold higher risk of *V. vulnificus* septicemia than healthy individuals [1,3].

The different contexts of *V. vulnificus* infection, in addition to the geographic range and pathogenicity of this organism, are used to classify *V. vulnificus* into three biotypes (Bt) [4,5]. In general, Bt1 causes sporadic cases of human skin infections and gastroenteritis and it is considered the most dangerous one [1], Bt2 causes both zoonotic cases of human skin infections and outbreaks of infection in fish [2,6], and Bt3 causes human skin infections associated with the handling of healthy tilapia (a spiny fish) [5,7]. In susceptible hosts, all three biotypes are thought to be able to spread from the skin or intestinal epithelium to the bloodstream, where they replicate rapidly and cause severe disease [8]. A recent phylogenomic analysis from the core genome of 80 *V. vulnificus* strains belonging to the three Bts demonstrates that this species is subdivided in five well-supported phylogenetic groups or lineages that do not correspond to Bts [9]. Lineage 1 comprises a mixture of clinical and environmental Bt1 strains, most of them involved in human clinical cases related to raw seafood ingestion. Lineage 2 accounts for a mixture of Bt1 and Bt2 strains from various sources related to aquaculture industry. Lineage 3 comprises exclusively all Bt3 strains, and Lineages 4 and 5 comprise a few Bt1 strains associated with specific geographical areas. This study also proposes a new updated classification of the species based on phylogenetic lineages as well as the inclusion of all Bt2 strains in a unique pathovar with the particular ability to cause fish vibriosis due to a transferable virulence plasmid (pathovar or pv. *piscis*) [10].

An eel (*Anguilla anguilla*) model of infection enabled study of mechanisms underlying the growth and pathogenesis of pv. *piscis* strains *in vivo* [2]. By using this model, it was demonstrated that *V. vulnificus* must overcome multiple host processes that constrain bacterial growth by using a series of virulence factors that promote survival within an aquatic host: i.e. the chromosomally-encoded O-antigen confers resistance to fish complement and a plasmid-borne gene encodes an outer membrane protein receptor for fish transferrin that contributes to growth in serum [11–13]. The *V. vulnificus* genes and processes related to human diseases are less well understood, due both to the greater genetic variability among *V. vulnificus* strains that induce pathology in humans and to variation in disease severity associated with patient immune status and blood-iron content [1]. Nevertheless, several *V. vulnificus* factors have been reported to play a role in human septicemia such as a potassium pump [14], a capsular polysaccharide (CPS) [15–18], a surface lipoprotein [19], the siderophore vulnibactin [16], the flagellum [20,21], the RtxA (or MARTX) toxin [22,23], and surface modifications by sialic acid moieties [24], all of which either contribute to growth in serum (potassium pump, siderophore…) or inflammation (CPS, lipoprotein, RtxA…) or immune evasion (RtxA, CPS..) or invasion (flagellum..).

To identify bacterial processes that enable *V. vulnificus* growth in human serum (HS) we undertook a forward genetic screen. Using an isolate of *V. vulnificus* capable of robust growth in HS, we first generated a nearly saturated transposon-insertion library and performed transposon-insertion sequencing to identify genes that are likely necessary for the *in vitro* growth of *V. vulnificus*. Comparative analyses that integrated these data with previously published results from transposon studies in other *Vibrio* species enabled us to define a set of core genes that multiple vibrios require for growth *in vitro*. Additionally, library sequencing following passage of the *V. vulnificus* mutant library in HS allowed us to identify genes that contribute to growth in this selective condition. To distinguish *V. vulnificus* genes that enable resistance to heat-labile antimicrobial factors from genes required to overcome the nutritional limitations, we developed an approach to integrate the results of parallel screens conducted in HS and heat inactivated HS. Our findings provide a comprehensive assessment of the bacterial factors required for *V. vulnificus* growth in HS and uncovered new genes required for CPS production in this human pathogen.

## Results

### *V. vulnificus* transposon library: generation and analysis

**Figure 1.**
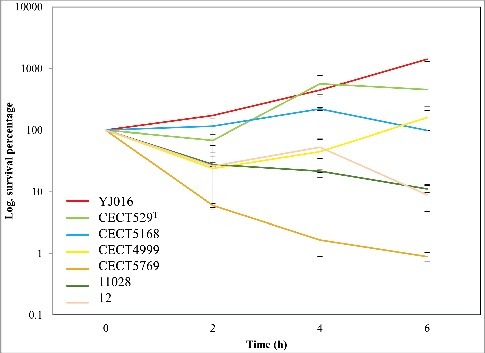
Resistance of *V. vulnificus* to HS. The initial bacterial population of the different *V. vulnificus* strains was considered “100” and survival at each time was calculated as a percentage. Bars indicate the standard deviation for three replicates. CECT, Spanish Type Culture Collection. Subspecific classification of the strains according to^9^: YJ016 and CECT5168 (or CDC-7184), formerly classified as Bt1, belong to Lineage 1, CECT529^T^ (or ATCC27562, type strain of the species), formerly classified as Bt1, belongs to Lineage 2; CECT4999 and CECT5769, formerly classified as Bt2 belongs to Lineage 2 and pv *piscis*; 11028 and 12, formerly classified as Bt3, belongs to Lineage 3.

**Figure 2.**
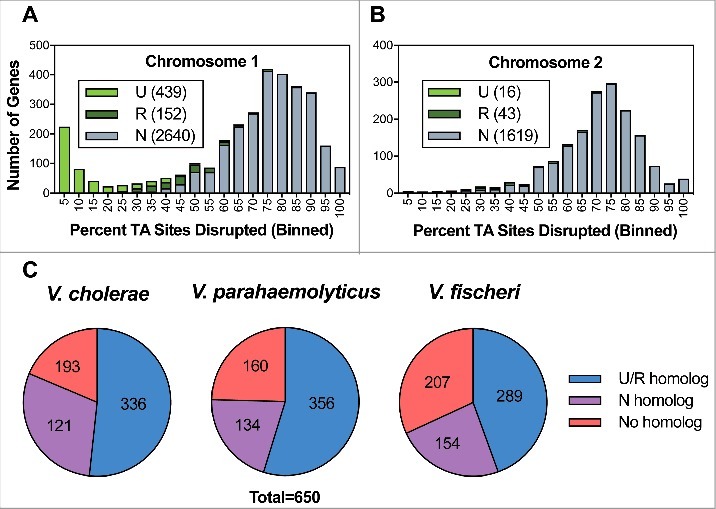
Characterization of the *V. vulnificus* transposon insertion library. Distribution of the percentage of TA sites disrupted per gene encoded on the large chromosome (A) and the small chromosome (B). Genes classified as underrepresented (U), regional (R), or neutral (N) are represented within each bin for each chromosome. (C) Status of genes homologous to a non-neutral *V. vulnificus* gene in *V. cholerae, V. parahaemolyticus*, and *V. fischeri.* U/R indicates underrepresented, regional, or comparably classified, homolog and N indicates neutral or comparably classified homolog.

We selected a *V. vulnificus* strain able to multiply in HS by assaying the viability in serum of a selection of isolates representative of the different phylogenetic lineages [9] (Table S1). The selected strain was YJ016 (Fig. 1), a blood isolate from a septicemic patient who ingested raw oysters in Taiwan [25]. Then, we implemented Transposon-Insertion Sequencing (TIS) [26] in YJ016 by adapting an established mutagenesis protocol [27]. We used conjugation to deliver the Mariner-based Himar1 transposon, which randomly inserts into TA dinucleotide sequences [28,29]. Our analysis revealed that the *V. vulnificus* mutant library contained at least 159,709 unique insertion mutants, corresponding to disruption of 75.9% of potential insertion sites. Genes were binned according to the proportion of TA sites disrupted per gene and these data were plotted for *V. vulnificus'* two chromosomes (Fig. 2AB). For both chromosomes, insertions were detected in >50% of potential insertion sites within most genes, with a modal insertion frequency of 70–80%, suggesting that 70–80% of non-essential insertion sites have sustained one or more insertion.

Approximately 300 genes encoded on *V. vulnificus'* large chromosome were disrupted in fewer than 10% of potential insertion sites, giving rise to a secondary peak within the distribution curve showing gene disruption frequency (Fig. 2A). This minor peak was absent from the small chromosome's distribution curve (Fig. 2B). Both observations are consistent with TIS-based studies of other *Vibrio* species, which report that the large chromosome harbours a disproportionately large number of essential genes relative to the small chromosome [27].

To identify the genes required for optimal *V. vulnificus* growth *in vitro*, we implemented the EL-ARTIST pipeline, which systematically identifies insertion mutants that are underrepresented among the pool of viable mutants that constitutes the transposon library. When all or most of the TA sites in a gene are infrequently disrupted, the gene is classified as “underrepresented” (U, in Fig. 2A,2B), and these genes are likely essential for *V. vulnificus* viability. When only a portion of a TA sites in a gene are infrequently disrupted, the gene is classified as “regional” (R, in Fig. 2A,2B), suggesting that part of the gene is essential for *V. vulnificus* viability. Finally, when few or none of the TA sites within a gene are infrequently disrupted, the gene is classified as “neutral” (N, in Fig. 2A,2B), and these genes are presumed to be dispensable for *V. vulnificus* viability. EL-ARTIST classified 455 chromosomal *V. vulnificus* genes as underrepresented and 195 genes as regional (9% and 4%, respectively) while the other 4259 chromosomal genes were classified as neutral (see Table S2, where these data are presented in full). Most of the non-neutral genes were encoded on the large chromosome; however, 59 were encoded on the small chromosome and 4 were encoded on a conjugative plasmid, pYJ016, which is dispensable for bacterial growth (Lien-I Hor, personal communication) (Fig. 2 and Table S2). Similar TIS data studies have shown that factors such as binding of the histone-like nucleoid structuring protein H-NS can lead to a low frequency of transposon-insertion in some non-essential genes [31]; consequently, the underrepresented and regional categories provide a comprehensive overestimate of the genes essential for the organism's growth *in vitro* [32]. This should be taken into account especially in case of the plasmid as extrachromosomal elements are the main targets for H-NS proteins [33].

To better understand the bacterial processes required for the growth of *V. vulnificus* relative to other *Vibrio* species, we compared the non-neutral *V. vulnificus* genes with similar TIS-based gene classifications from *V. cholerae, V. parahaemolyticus*, and *V. fischeri* [27,32,34]. The 650 chromosomally-encoded non-neutral *V. vulnificus* genes showed greatest concordance (i.e., non-neutral classification in both data sets) with *V. parahaemolyticus* (356), followed by *V. cholerae* (336) and *V. fischeri* (289) (Fig. 2C). However, these comparisons should be interpreted with caution because of methodological biases that could reflect evolutionary relatedness (e.g., *V. vulnificus* and *V. parahaemolyticus* encode more genes than *V. cholerae* and *V. fischeri*). Organism-specific gene duplications and speciation events that yielded functionally redundant genes lacking sequence homology may also account for species-specific results.

Despite such potential confounding factors, 253 genes were concordantly classified as non-neutral across the 4 species, and therefore represent a TIS-defined set of *Vibrio* ‘essential’ genes. The list of concordant genes, along with their functional categorization (based on ‘Gene Ontology’ obtained via Uniprot [35) and discrepancies identified from individual comparisons of each species against *V. vulnificus*, are reported in the supplemental materials (Table S3 and S4).

Since the plasmid pYJ016 is not found in *V. parahaemolyticus, V. cholerae* or *V. fischeri*, the classification of 4 plasmid-encoded genes as non-neutral in *V. vulnificus* was somewhat unexpected. Two of these genes, *VVP32* and *VVP66,* encode putative addiction module antitoxins, and thus are likely involved in plasmid maintenance. A low frequency of transposon insertion attributable to H-NS binding could explain why the other two non-neutral plasmid genes, *VVP39,* a putative PilT protein, and *VVP67*, a hypothetical protein, were classified as underrepresented.

We also compared the *V. vulnificus* gene classifications and the 253 non-neutral genes observed across all 4 *Vibrio* species to the set of essential genes from *E. coli* K-12, in order to identify genetic requirements for bacterial growth that differ between vibrios and other genera. *V. vulnificus* homologs of 31 essential *E. coli* genes were classified as neutral in our analysis. Of these, 20 were found to be duplicated in the *V. vulnificus* genome, which likely accounts for the dispensability of individual homologs for the strain's *in vitro* growth. The remaining 11 genes (Table S5) were identified as discordant in previously published comparisons of the essential *E. coli* genes to *V. cholerae* and *V. parahaemolyticus* genes classified as neutral [27,32], suggesting that these genes' lack of essentiality is a common feature that distinguishes vibrios from *E. coli*. These genes include riboflavin biosynthesis and chromosome compaction genes, some of which Chao *et al.* confirmed are dispensable for the viability of *V. cholerae* [27]. Future studies can investigate how such processes differentially contribute to the viability of *E. coli* and vibrios and how their cellular roles have diverged among these gamma-proteobacteria.

### Identification of *V. vulnificus* genes required for survival/growth in HS

**Figure 3.**
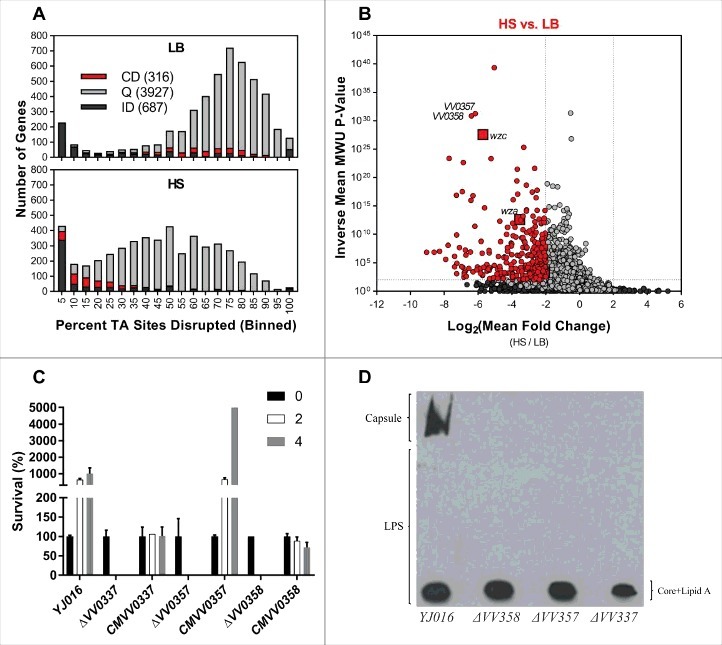
Identification of genes conditionally depleted in human serum (A and B) and characterization of mutants in selected genes (C y D). (A) Distribution of the percentage of TA sites disrupted per gene in the LB or HS-selected libraries. Genes classified as conditionally depleted (CD), queried (Q), or insufficient data (ID) are indicated in each bin. (B) Results of Con-ARTIST analysis. x-axis indicates change in relative abundance of insertion mutants per gene between LB and HS, and the y-axes indicates the concordance of independent insertion mutants within each gene. Genes exhibiting a greater than 4-fold change (Log_2_(mean fold change) <- 2 or > 2) across multiple mutants (mean inverse P-value > 10^2^) are considered conditionally depleted and are shown in red. Large red squares indicate *wza* and *wzc*, which are known to be attenuated in HS. (C) Survival percentage of YJ016, the deletion mutants and its complemented strains after 2 and 4 h incubated in human serum. (D) CPS detection by immunostaining. Bacteria were grown in LB (1% NaCl), associated-cell polysaccharides were extracted, separated by SDS-PAGE, transferred to NC-sheets and immunostained with specific antibodies against YJ016.

We used TIS to identify genes involved in resistance to the multiple heat sensitive (i.e. complement proteins) and heat stable (nutritional constraint) anti-bacterial factors present in HS [36]. First, we optimized screening conditions by generating a mutant unable to synthesize capsular polysaccharides by in-frame deletion. The selected gene was *wza*, a gene encoding an outer membrane protein involved in the CPS translocation [37,38]. As expected, the mutant *∆VV0337/∆wza* exhibited phenotypes indicative of capsule deficiency (translucent colonies on agar plates and sedimentation/aggregation in the bottom of culture tubes) (Fig. S1). The mutant was incubated in HS and we found that no cfu were recovered after 2h incubation (Fig. 3C). Consequently, we determined that 2 h incubation would be sufficient to select against mutants unable to survive/grow within HS.

Then, we compared the relative abundance of insertion mutants in the transposon library when this was incubated in HS vs. LB (Fig. 2 and 3). We binned genes from both chromosomes according to the proportion of TA sites disrupted per gene and plotted these data for the LB and serum populations (Fig. 3A). Comparison of the LB and HS distributions revealed that the large population of genes with insertions in a high proportion of TA sites in the LB library underwent a substantial left-shift in the HS-selected library. This trend is consistent with a genotype-independent population constriction (i.e., an experimental bottleneck) that occurred during exposure to HS. We implemented a modified version of the Con-ARTIST pipeline, which performs simulation-based normalization to model and compensate for the stochastic effects of an experimental bottleneck [30], to compare the abundance of individual mutants in the LB and HS-selected libraries (Table S6). Importantly, our modified analysis integrates data from independent insertions across the length of each gene in an additional effort to minimize the impacts of experimental noise. Thus, our analysis was restricted to the 4243 genes with 5+ independent insertions; 687 did not meet this criterion and are classified as “insufficient data”, (ID) in Fig 3A. 316 genes met criteria used to define the loci required to survive/grow in HS (Log_2_(mean fold change) < -2, inverse mean MWU *P*-value > 100; Fig. 3B, genes shown in red); these genes were classified as conditionally depleted (CD).

As expected, these genes included the control gene *wza* (*VV0337)* together with *wzb* (*VV0339*) and *wzc* (*VV0340*) (Fig. 3B, large squares), whose homologs also encode proteins involved in the translocation of CPS type 1 polysaccharides (CPS-1) in *E. coli* and *V. vulnificus* (PMC403799) [37,39–41], as well as numerous (23) additional genes that likely mediate lipopolysaccharide (LPS) and CPS biosynthesis and transport. Identification of genes previously linked to capsule biogenesis and sensitivity to HS in other strains [37–42] provides confidence that our screening approach and analysis is sufficient to identify genes required for survival/growth in HS. Genes outside of the CPS locus that were found to be required for growth in HS include loci predicted to encode ABC transporters, mediators of succinate metabolism, Clp protease, genes for nucleotide biosynthesis, the virulence K^+^ related transporter (*trkA*), the membrane protein OmpH, the TolBARQ membrane system, the type 2 secretion system (T2SS) and the Na^+^ transporting NADH-ubiquinone reductase (NQR) complex.

To validate the results of our screen, we selected 2 genes – *VV0357* and *VV0358* – for further study. Both genes were classified as conditionally depleted following exposure to HS (Fig. 3B), but neither were previously associated with survival in HS. We generated in-frame deletions of each gene and assessed the survival/growth of these single-gene deletion mutants in HS relative to wild type *V. vulnificus* and the *ΔVV0337* mutant (Fig. 3C). Both the *ΔVV0357* and *ΔVV0358* mutants were as sensitive as the *ΔVV0337* mutant to HS; no colonies were recovered after 2 h incubation in HS (Fig. 3C). In addition to serum-sensitivity, the *∆VV0357* and *∆VV0358* strains exhibited phenotypes consistent with capsule-deficient mutants, including agglutination in culture tubes and translucent colony morphology (Fig. S1). Given the importance of the capsule for *V. vulnificus* growth *in vivo*, we tested the virulence of the mutant strains for mice (iron-overloaded model) and found that all three mutants were avirulent (Table 1). Complementation with plasmid-based expression of the deleted genes was sufficient to restore wild type phenotypes to the mutant strains (Fig. S1 and Table 1), although the complemented strains survived in HS without multiplication (Fig. 3C). Therefore, we hypothesized that *VV0357* and *VV0358,* like *wza* homologue *VV0337*, are involved in capsule biosynthesis. The absence of capsule production in the *VV0357* and *VV0358* mutants was confirmed via immunoblotting: no CPS was observed in the surface polysaccharide fractions from Δ*VV0357* and Δ*VV0358* (Fig. 3D). Collectively, these results indicate that *VV0357* and *VV0358* encode proteins that are necessary for capsule formation, HS-resistance, and virulence in mice.

**Table 1.** Virulence assays on iron-overloaded BALB/C mice.

Strain	LD_50_ (cfu/mouse)^*^
YJ016	1 ± 0.5 × 10^2^
*ΔVV337*	>10^7^
*CMVV337*	2.5 ± 5 × 10^2^
*ΔVV357*	>10^7^
*CMVV357*	3 ± 2 × 10^3^
*ΔVV358*	>10^7^
*CMVV358*	7 ± 2 × 10^3^

* average and standard deviation from three different experiments.

*VV0357* and *VV0358* are annotated as a “zinc-binding dehydrogenase” and a “hypothetical protein” respectively, and both genes are located in relative proximity to the annotated CPS gene cluster (Fig. S2). However, homology searches revealed *VV0358* and *VV0357* are not widespread among *V. vulnificus* and only the strain CG64, an environmental isolate recovered in geographic and temporal proximity to the YJ016 [43], encodes a *VV0358* homolog, raising the possibility that this gene was acquired by horizontal transmission (Fig. S2). Phyre2-based structural homology analyses suggest that *VV0357* encodes a protein with two transmembrane helices potentially implicated in protein translocation and that *VV0358* encodes a protein with strong structural homology to a heparinase III protein (Fig. S3). BLASTP queries also identified a putative heparinase II/III-like domain (pfam07940) in VV0358.

### Comparison of the genes required for growth in HS and hiHS

**Figure 4.**
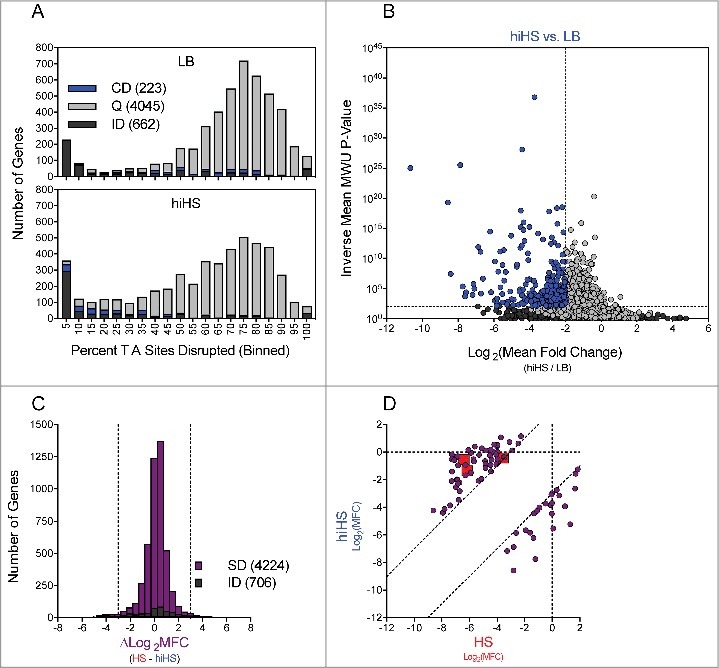
Identification of genes conditionally depleted in heat inactivated human serum. (A) Distribution of the percentage of TA sites disrupted per gene in the LB or hiHS-selected libraries. Genes classified as conditionally depleted (CD), queried (Q), or insufficient data (ID) are indicated in each bin. (B) Results of Con-ARTIST analysis. x-axis indicates change in relative abundance of insertion mutants per gene between LB and HS, and the y-axes indicates the concordance of independent insertion mutants within each gene. Genes exhibiting a greater than 4-fold change (Log_2_(mean fold change) <- 2 or > 2) across multiple mutants (mean inverse P-value > 10^2^) are considered conditionally depleted. (C) The difference in Log_2_(Mean Fold Change) (HS – hiHS) was plotted for SD (sufficient data: 5+ informative sites in both comparisons) and ID (insufficient data: <5 informative sites in one or more comparisons) genes. (D) Log_2_(Mean Fold Change Values) from the HS and hiHS samples are plotted on the x- and y-axes, respectively, for genes with >8-fold difference between the two selections. Large red squares indicate *wza, VV0357*, and *VV0358*, which were conditionally depleted in HS but not in hiHS.

We analysed the relative abundance of insertion mutants in the *V. vulnificus* transposon library when this was incubated in heat inactivated HS (hiHS) to discriminate between genetic factors that protect against heat sensitive vs. heat labile constraints. Again, we binned genes from both chromosomes according to the proportion of TA sites disrupted per gene (Fig. 4A) and compared these data for the LB and hiHS populations. The left-shift in the large population of genes disrupted in a high proportion of TA sites was minimal compared to that observed in the HS-selected library, indicating that the experimental bottleneck was less severe in the hiHS screen. Using Con-ARTIST, we compared the abundance of individual mutants in the LB and hiHS-selected libraries (Table S7), with analysis restricted to the 4268 chromosomal genes with 5+ independent mutations, and 662 genes classified as “insufficient data”. 223 genes met criteria used to define the genes required to survive/grow in hiHS (Log_2_(mean fold change) < -2, inverse mean MWU *P*-value > 100) (blue genes in Fig. 4B). Thus, fewer genes are classified as conditionally depleted following exposure to hiHS than HS. Notably, none of the *wza-wzb-wzc* genes found to be conditionally depleted in HS were conditionally depleted in hiHS, which indicates, as expected, that the components of serum (such as complement) that prevent the survival/growth of capsule deficient mutants are heat sensitive. Several genes that encode or are predicted to encode ABC transporters, Clp protease, succinate metabolism, TolBARQ, TrkA K^+^ transporter, OmpH membrane protein, T2SS, and the NQR *system* were found to be conditionally depleted in hiHS as well as in HS. Thus, we speculate that these genes contribute to processes required for growth in HS independently of the heat-labile antimicrobial properties of HS.

To integrate the results of our parallel screens in HS and hiHS, we compared each gene's Log_2_(MFC) values (vs. LB, as shown in Figs. 3B and 4B), which are a reflection of fitness in the selective condition, for the parallel screens. To minimize the effects of noise, we restricted this comparison to the 4224 genes with 5+ independent mutations in both the HS and hiHS analyses (706 genes did not meet this criterion and are classified as “insufficient data”) (Fig. 4C). Most genes showed minimal difference between the two screens (-3 < ∆Log_2_MFC < 3), however 83 genes showed a > 8-fold discrepancy between the two screens and were classified as “divergent” (the 2 tails in Fig 4C) (Table S8-S9). To highlight the source of disparity between the HS and hiHS screens, we plotted the Log_2_MFC values observed in HS and hiHS on the x- and y-axes, respectively (Fig. 4D). Genes in the lower right of the graph exhibited more severe attenuation in hiHS than in HS, while genes in the upper left of the graph, including *VV0337, VV0357, VV0358*, and other components of the CPS locus, exhibited more severe attenuation in HS than in hiHS. Apart from the CPS locus, a number of additional genes, including 32 predicted to encode hypothetical proteins (Table S8), without clear associations with CPS biosynthesis, were also found to be more severely attenuated in HS than in hiHS. Further studies are necessary to determine whether these genes encode previously undescribed mediators of CPS biosynthesis or factors that contribute to additional processes that fortify bacterial growth in the presence of heat-labile antimicrobial factors.

While many heat-labile components of HS are directly bactericidal, the nutrient content of HS also imposes a restriction on bacterial growth [36]. We hypothesized that many of the mutants found to be similarly attenuated in the HS and hiHS screens had a reduced capacity to overcome the nutritional constraints of HS (Table S9). 110 genes were found to be conditionally depleted in both screens to a comparable degree (-3 < ∆Log_2_MFC < 3), including all 6 components of the Na^+^ transporting NADH-Ubiquinone reductase (NQR) complex (Fig. S4). The NQR complex is a membrane pump that creates an electrochemical gradient to drive uptake of diverse substrates (including several amino acids, citrate, and inorganic phosphate), and efflux of antibiotics in *V. cholerae* [44]. In addition to the NQR complex, mutants in 7/12 components of the type II secretion system (Fig. S4) were comparably attenuated in both conditions. *V. vulnificus* utilizes the T2SS to secrete a number of proteins, including VvpE, a protease that degrades haemoglobin, and VvhA, a hemolyisn [45] and these secreted products may facilitate nutrient acquisition. Moreover, both Clp protease subunits, the potassium transporter TrkA, 10 dehydrogenases, a TolBARQ system and a series of genes involved in nucleotide metabolism (*VV0625, VV0752, VV0775, VV0776 and VV0843*) were also attenuated when YJ016 was grown either in HS or hiHS.

### Discussion

Here we report the first application of saturating transposon mutagenesis to study the opportunistic pathogen, *V. vulnificus*. Using TIS, we identified genes that likely enable the growth of *V. vulnificus* in rich media, in addition to those that allow this organism to overcome impediments to bacterial growth intrinsic to HS. By integrating our analysis of these *in vitro* data sets with previously reported transposon studies conducted in related vibrios and with an additional screen conducted in parallel, we offer a strategy to: 1) identify genes required for the growth of multiple *Vibrio* species, and 2) distinguish genes that are required to resist the heat-labile antimicrobial activity of HS, mediated by complement and other protein factors, from genes that enable *V. vulnificus* growth or survival in HS even in the absence of heat-labile antimicrobial activities.

Integrating our observations gleaned from analyses of the *V. vulnificus* transposon-insertion library grown in rich medium with the findings from 3 previous similar studies conducted in related *Vibrio* species revealed that 253 genes are likely required for the *in vitro* growth of all 4 *Vibrio* species. While further studies are necessary to validate and curate this list, these 253 genes represent the best available estimation of the *in vitro* essential gene set of members of the *Vibrio* genus. The majority of core *Vibrio* “essential” genes are essential for the *in vitro* growth of *E. coli* K-12 as well, yet we also identified a number of discordantly classified genes. Many of these discrepancies are apparently attributable to gene duplication events; however others (e.g., riboflavin biosynthesis) constitute verifiable differences in the genes necessary for the growth of vibrios relative to *E. coli*. Thus, our approach, which highlights differentially required growth processes, could facilitate future studies of the genetic divergence of these gamma proteobacteria.

Through our screens of *V. vulnificus* growth in HS, we identified *VV0357* and *VV0358*, which are 1) genetically linked to the capsular polysaccharide biosynthesis gene cluster specifically in *V. vulnificus* YJ016, 2) dispensable for growth in rich medium, and 3) required for growth in HS, but not in hiHS. Additional studies, utilizing in-frame deletions of the *VV0357* and *VV0358* genes, validated our screen and revealed that *VV0357* and *VV0358* are necessary for production of the *V. vulnificus* capsule. These findings were further supported by observations that *VV0357* and *VV0358* display phenotypes consistent with capsule-deficient mutants, including translucent colony morphology, serum sensitivity and avirulence for mice. Although the role of capsule in resistance to serum has been previously reported in *V. vulnificus*, most of this research was performed with spontaneous translucent variants [16-18]. Regarding the role of both genes in capsule biosynthesis, *in silico* analyses suggest that VV0357 and VV0358 may act as a membrane dehydrogenase and a heparinase II/III-like protein, respectively. Heparinases II and III cleave at the linkages between hexosamine and uronic (or derivatives) acid residues in heparan sulfate, yielding mainly disaccharides. Considering that bacteria with related-CPS can use lyases to degrade molecules aberrantly released to the periplasm [46,47], we propose that CPS of *V. vulnificus* YJ016 may be a heparosan/alginate-like polysaccharide. Nevertheless, this would be a rare event in the species since only one genome (from strain CG64) among the total ones analysed contained a homologous gene. This genome corresponds to a *V. vulnificus* strain isolated from the environment in the same geographical area than YJ016 [43]. This isolate was further analysed and resulted to belong to the same lineage than YJ016 (Roig et al., unpublished). In addition, this isolate was positive for all the genetic markers for human virulence described in *V. vulnificus* [48]. The existence of a specific group of Taiwanese clones presenting a heparosan/algynate-like polysaccharides on their surfaces should be confirmed by chemical analysis of the CPS.

The comparison of the relative abundance of insertion mutants in the HS and hiHS libraries allowed us to discover the genes that the bacterium would use to overcome nutritional constrain in HS. We found evidence that *V. vulnificus* YJ016 could respond to the restriction in purines and pyrimidines in serum supporting Samant et al study [49], by producing its own biosynthetic enzymes, as well as to the absence of simple carbon and nitrogen sources by using T2SS to secrete exoenzimes that would degrade complex macromolecules, such as proteins, present in serum. The genes encoding the NQR system, which maintains the Na^+/^proton gradient in certain natural environments [50], were also found to be required for growth in both HS and hiHS. We speculate that the NQR system could help to maintain the electrostatic equilibrium in a stressing medium such as HS. This conclusion is further supported by reports that the NQR system maintains the proton-motive force when bacterial membranes are altered [44], and by our finding that genes necessary either for resistance to bactericidal peptides (e.g., *ompH*) [51] or for LPS modifications that also promote resistance to these peptides [52], were required for growth/survival in HS and hiHS. Similarly, the peripheral membrane protein TrkA, an essential member of the potassium transporter was described as essential for YJ016 to grow in HS and hiHS [14]. Finally, and in contrast to previous reports [24], the sialic acid genes were not found relevant for serum growth in the present study.

In summary, our TIS-based studies yielded many observations worthy of future investigation and illustrate the potency of TIS-based analyses to dissect complex phenotypes like pathogen growth in HS. When applying this technique to *V. vulnificus*, a dangerous septicaemic pathogen, we have been able to describe new genes involved in protection against human complement from new CPS biosynthetic genes to genes for hypothetical proteins as well as genes with a putative role in overcoming heat-resistant constraints in serum such as genes for OMPs (OmpH and others), biosynthetic/modifying LPS enzymes, a NQR system, a T2SS, and biosynthetic nucleotide enzymes, all of them supposedly essential to grow in serum and to allow the pathogen to proliferate in blood and cause death by septicemia.

## Material and methods

### Strains, plasmids and general culture conditions

Strains and plasmids are listed in S1 Table. All the strains were routinely grown in Luria-Bertani broth (LB+0.5% NaCl) with shaking (200 rpm) or on LB-agar (LBA) at 37 ºC for 18–24 h. A spontaneous streptomycin resistant mutant (YJSm^r^) was selected after growing the strain YJ016 on LBA-Sm (streptomycin, 500 µg/ml) and checking that the mutant and the wild-type's growth curves in LB were statistically similar. YJSm^r^ was used as the recipient strain in conjugation experiments with the Tn-donor strain and is the parental strain for all knockout mutants in the genes selected after TIS assays. Antibiotic concentrations used in the experiments for genetic modification were: Sm, 500 µg/ml; chloramphenicol (Cm), 20 µg/ml (*E.coli)* or 2 µg/ml (*V. vulnificus)*; and kanamycin (Km), 150 µg/ml. *E. coli* SM10 *lambda pir* carrying the Himar1 suicide Tn vector pYB742 (CmR) was used for Tn mutagenesis [53]. All strains were kept in LB+ 20% glycerol at -80 ºC.

### Resistance and growth in HS

Cells in exponential phase of growth in LB were washed twice in PBS and were inoculated in HS (commercial HS, Sigma, H4522) or heat-inactivated HS (56 ºC, 30 min) at 10^4–5^ cfu/ml by triplicate. Tubes were incubated at 37ºC with shaking (170 rpm) and bacterial growth was monitored by plating on LBA plates at 0, 2, 4 and 6 h post inoculation. (Human complement starts to inactivate spontaneously after 6–8 h of incubation at 37ºC; unpublished result). Serial ten-fold dilutions in PBS (phosphate buffered saline, pH 7) were spotted (10 µl) on LBA plates (drop plate count). Plate counting was performed by triplicate.

### Tn library construction

The Mariner-based Himar1 Tn (which inserts into TA sites) and a previously published protocol [26,30] were used to construct two independent libraries for each condition assayed. Few changes were made in order to optimize the saturation of our transposon mutant library. An initial Tn-library was created in LBA by mixing washed bacteria from 1 ml of an overnight culture of Tn donor and receptor (Table S1) in a final volume of 500 µl of fresh LB at a ratio of 1:1. Then, 50 µl-aliquots were spotted onto 0.45 µm-filters (Millipore, HAWP04700) placed on LBA plates that were incubated for 6 h. After conjugation, the filters were recovered and grown bacteria were spread onto LBA+Sm+Km plates of 24.5 × 24.5cm [2] (Corning) that were incubated at 37ºC for 24 h.

Bacteria grown on the plates (approx. 1.5 million) were scrapped with 12 ml of fresh LB and 3 ml were used to obtain genomic DNA exactly as Chao *et al.* described [26,27] and the rest (around 20 ml) was kept with 20% glycerol at -80 ºC. DNA from this library was used for determination of genes required for *V. vulnificus* growth in vitro

### Library selection

The obtained Tn-library was again inoculated in LB (control) and, in parallel, in HS (tested condition 1) and inactivated-HS (tested condition 2) at a ratio 1:30 vol/vol (approximately 10^8^ cfu/ml) and incubated at 37ºC for 2 h. Cells were recovered by centrifugation, resuspended in 3 ml of LB and immediately spread on LBA+Sm+Km plates (24.5 × 24.5cm [2, Corning) that were incubated at 37ºC overnight. Bacteria were recovered and DNA genomic was obtained as explained before. A volume of around 20 ml of bacterial suspension was separately kept with 20% glycerol at -80 ºC.

### Mapping and analysis of transposon insertion sites

5–10 µg of genomic DNA was fragmented into pieces of ≈350 bp by sonication (M220 focused ultrasonicator, Covaris, Woburn, MA), then DNA ends were repaired with the Quick Blunting™ Kit (NEB, E1201L), the Tn junctions were amplified, adaptor sequences were attached and each Tn-library sequenced on a MiSeq (Illumina, San Diego, CA) [26,27].

Reads of more than 15 nucleotides were mapped to the *V. vulnificus* YJ016 genome, which is composed of two chromosomes (chromosome 1, NC_005139.1; and 2, NC_005140.1) and one plasmid (pYJ016, NC_005128.1) [25]. We discarded reads that did not align to any TA site in the genome, and we randomly distributed the reads mapping to multiple TA sites. The number of reads at each TA site was tallied, datasets were normalized for origin proximity, and Tn-insertion profiles were depicted using Artemis software [54]. Then, EL-ARTIST software [30] was used to analyze the two chromosomes and the plasmid pYJ016, independently. ARTIST uses simulation-based normalization to model and compensate for experimental noise, and thereby enhances the statistical power in conditional TIS analyses. The statistical models and analysis to provide robust significance of the results are detailed in [30]. Chromosome 1 classifications were obtained using hidden Markov model analysis following usual values for sliding window (SW) training (10 TA sites; P < 0.005) while chromosome 2 classifications were obtained using 15 TA sites. For chromosome 1 and plasmid, genetic loci with fewer than 10 TA sites were retroactively excluded from classification. In chromosome 2, at least 15 TA sites were used.

Because of the differences in TA % disrupted between Tn-libraries, we used Mann-Whitney test in order to compare the TA abundances. Minimum 90% Mann-Whitney U (MWU) test values, p-value<0.00001 and fold change (FC) bigger than 5 were considered for the analysis. The FC represents the number of times a gene is more represented in one Tn-library vs*.* other. Considering the TA positions that could have been disrupted and the TA positions covered in our Tn-library, we classified genomic loci as “under-represented”/“regional” when the number of sequenced transposons in the loci was significantly low, or “neutral”, when reads were mapped in practically the total sequence of the gene. Further, we differentiated two categories within essential genes on the basis of the location of reads, “under-represented” and ”regional” when the reads were located in the full sequence or in a part of the gene, respectively.

### Bioinformatic analyses

Genes listed as under-represented for survival/growth in HS were subject to additional bioinformatic analyses; in particular, genes annotated as hypothetical proteins were re-annotated using HHpred or BLASTP against Genbank. Moreover, domains were analysed using the Conserved Domain Database (CDD) on the NCBI website. The Protein homology/analogy recognition engine V 2.0 (Phyre [2) pipeline was used for analysis of the predicted VV0358 amino acid sequence [55]. Primers were designed manually using basic sequence edition programs, mainly ApE (A plasmid Editor v2.0.47;http://biologylabs.utah.edu/jorgensen/wayned/ape/).

The BLASTP tool was used to check for the presence of a genes found to be under-represented in *V. vulnificus* YJ016 in *V. cholerae* N16961, *V. parahaemolyticus* RIMD 2210633, V. fischeri ES114, *Moraxella catarrhalis* BBH18, *E. coli* ST131 and *E. coli* K12. In these comparisons, we only considered hits that had at least 50% of identity, covered 85% of the predicted polypedtide sequence and had a p-value was smaller than 10^−5^. Genes of interest were functionally classified using Uniprot [35].

### Deletion mutants and complementation

Mutants (Table S10) were obtained using allelic exchange [56]. Mutants were constructed by ligating ≈500 bp PCR-products, generated from the primers in Table S10, to the suicide vector, pDM4, using Gibson assembly [57]. Allele exchange vectors were transferred from *E.coli* SM10 to YJSm^r^ by conjugation.

To carry out the conjugation, 40 µl of washed overnight cultures of each bacterium were mixed and spotted on 0.45 µm-filter (Millipore, HAWP04700). After 4h of incubation, cells were recovered in LB and spread on LBA+Cm+Sm. Finally, sucrose-based counter-selection was performed as described by Donnenberg and Kaper [56]. Briefly, 3 random Cm+Sm resistant colonies were grown overnight, the culture was diluted 1:100 in LB+10% sacarose (wt/vol) and was incubated again until it reached a value of Abs_600_ = 0.5. Then, 100 µl of eachone of a serial ten-fold dilutions of the culture were spread on LBA plates. Colonies were checked by PCR and DNA sequencing to confirm the deletion was in frame, and the mutants were kept in LB+ 20% glycerol at -80 ºC.

Complementation of the mutants was done using derivatives of pMMB207 (Cm^r^). The selected genes were amplified by PCR (S10 Table). PCR purified products and the pMMB207 were digested with two different FastDigest enzymes (Thermo Scientific). Plasmid was treated with FastAP Thermosensitive Alkaline Phosphatase (Thermo Scientific, EF0652) and ligated to the gene with T4 ligase (Thermo Scientific). Transformed *E. coli* SM10 cells (same protocol as deletion mutants) were conjugated with the corresponding deletion mutant using the same protocol described before. Colonies were checked to confirm the gene was inserted in frame, and the complemented mutants were kept in LB+ 20% glycerol at -80 ºC until used.

### Phenotypic characterization of the mutants

#### a) Animal model of sepsis: mean lethal dose determination (LD_50_)

The bacterial virulence for mice of the wild-type and each one of the selected mutants was determined in 6- to 8-week old female (mice BALB/c, Charles River, France) by using the iron-overloaded model of infection according to [58]. This model consists in pre-inoculating mice with 2 μg of iron per g of body weight as hemin 2 h prior to the infection, and then, intraperitoneally injecting animals with ten-fold serially diluted bacterial suspensions in PBS. The LD_50_ was calculated as described [59].

#### b) Growth in serum

The growth of the mutants with regard to the wild-type strain was tested in the following media: HS, HIS, Inoculated media (10^5^ cfu/ml) were incubated for 6 h at 37ºC, samples were taking at 0, 2 and 6 h post-incubation for bacterial counting on LBA plates by drop plate [60].

#### c) Surface antigen analysis

Crude fractions of cell-associated polysaccharides were obtained from overnight cultures in LB essentially as described by Hitchcock and Brown [61] and Valiente [12]. Polysaccharides were separated by SDS-PAGE [62] in discontinuous gels (4% stacking gel, 10% separating gel), transferred to a PVDF membrane (Bio-Rad) [63] and subjected to immunoblot analysis. The membranes were stained with a rabbit anti-inactivated YJ016 cells serum especially enriched in antibodies against capsular antigens (anti-capsular serum) [64]. The serum was diluted 1:3000 and membranes were developed following incubation with anti-rabbit IgG HRP-conjugated secondary antibody diluted 1:10000 (Sigma), using Immobilon Western Chemiluminescent HRP Substrate (Millipore).

## Supplementary Material

Suppl_mat_Comprehensive_identification_of_Vibrio_vulnificus_genes.zip

## Statistical analysis

All the experiments were performed by triplicate. Statistical analysis was performed using SPSS 17.0 for windows. The results are presented as means ± SE (standard error of the means). The significance of the differences between means was tested by using the unpaired Student´s t-test with a *P* < 0.05. When the effects of more than two independent variables were taken into account, the results were analyzed by variance analysis ANOVA followed by Tukey´s test.
