# Supplementary material for: Comprehensive identification of Vibrio vulnificus genes required for growth in human serum
Source: Virulence. 2018 Jun 21;9(1):981–93. doi: 10.1080/21505594.2018.1455464 (PMC6037467; doi:10.1080/21505594.2018.1455464)
Supplement: Suppl_mat_Comprehensive_identification_of_Vibrio_vulnificus_genes.zip [file kvir-09-01-1455464-s001.zip › Suppl_mat_Comprehensive identification of Vibrio vulnificus genes/figuressup.pptx]

## Slide 1
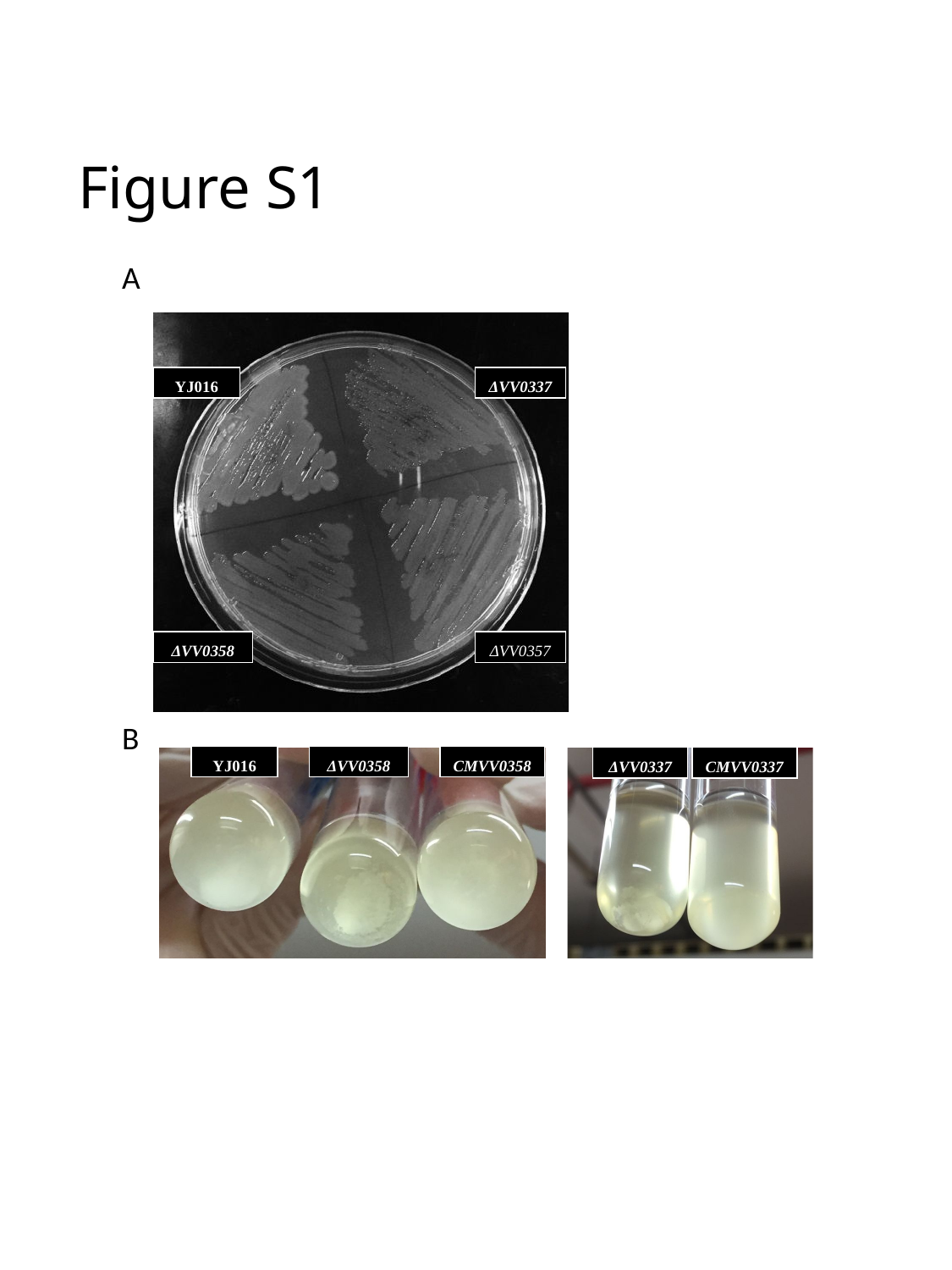

# Figure S1
A
YJ016
ΔVV0337
ΔVV0358
ΔVV0357
B
YJ016
ΔVV0358
CMVV0358
ΔVV0337
CMVV0337

## Slide 2
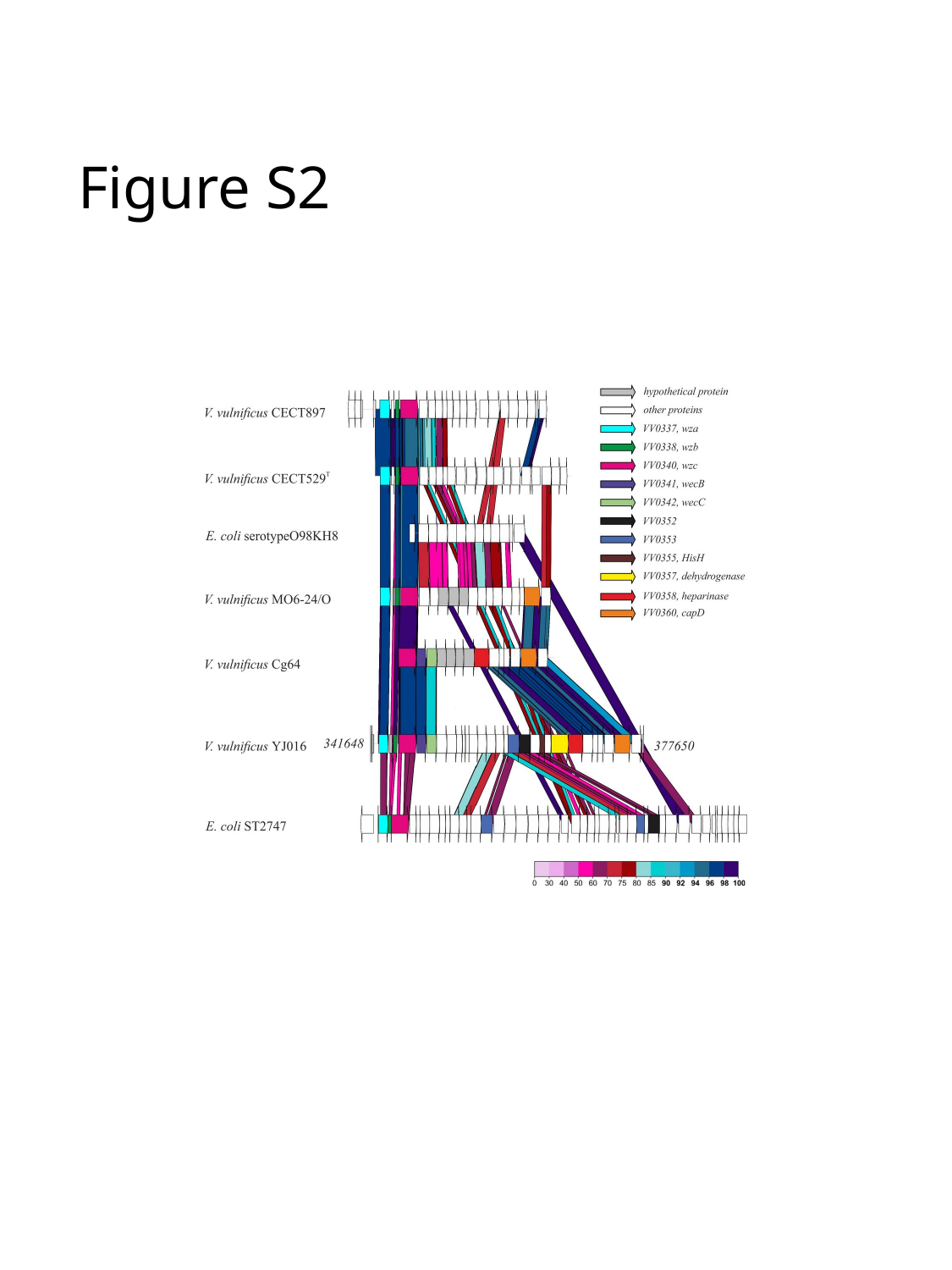

# Figure S2

## Slide 3
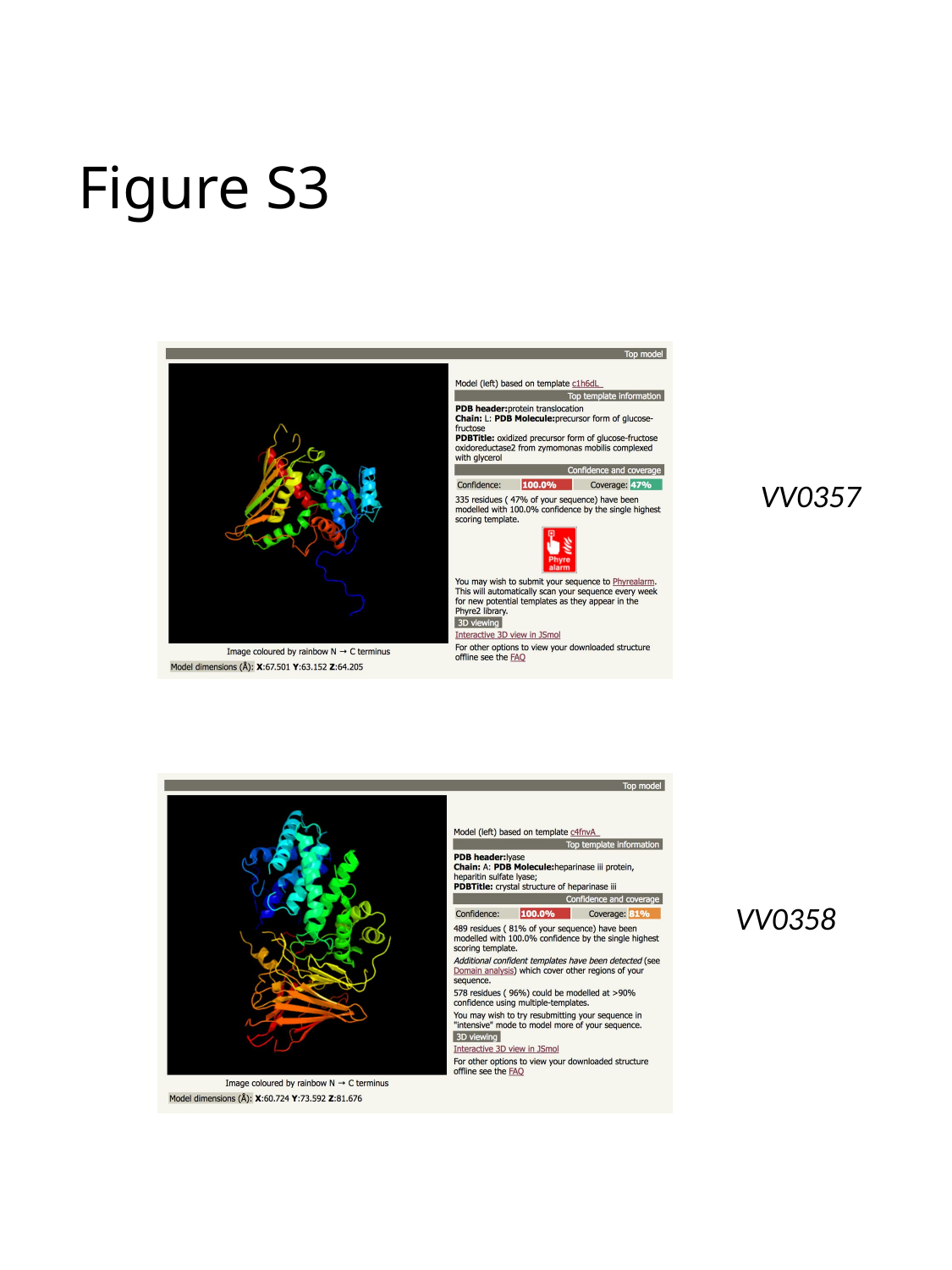

# Figure S3
VV0357
VV0358

## Slide 4
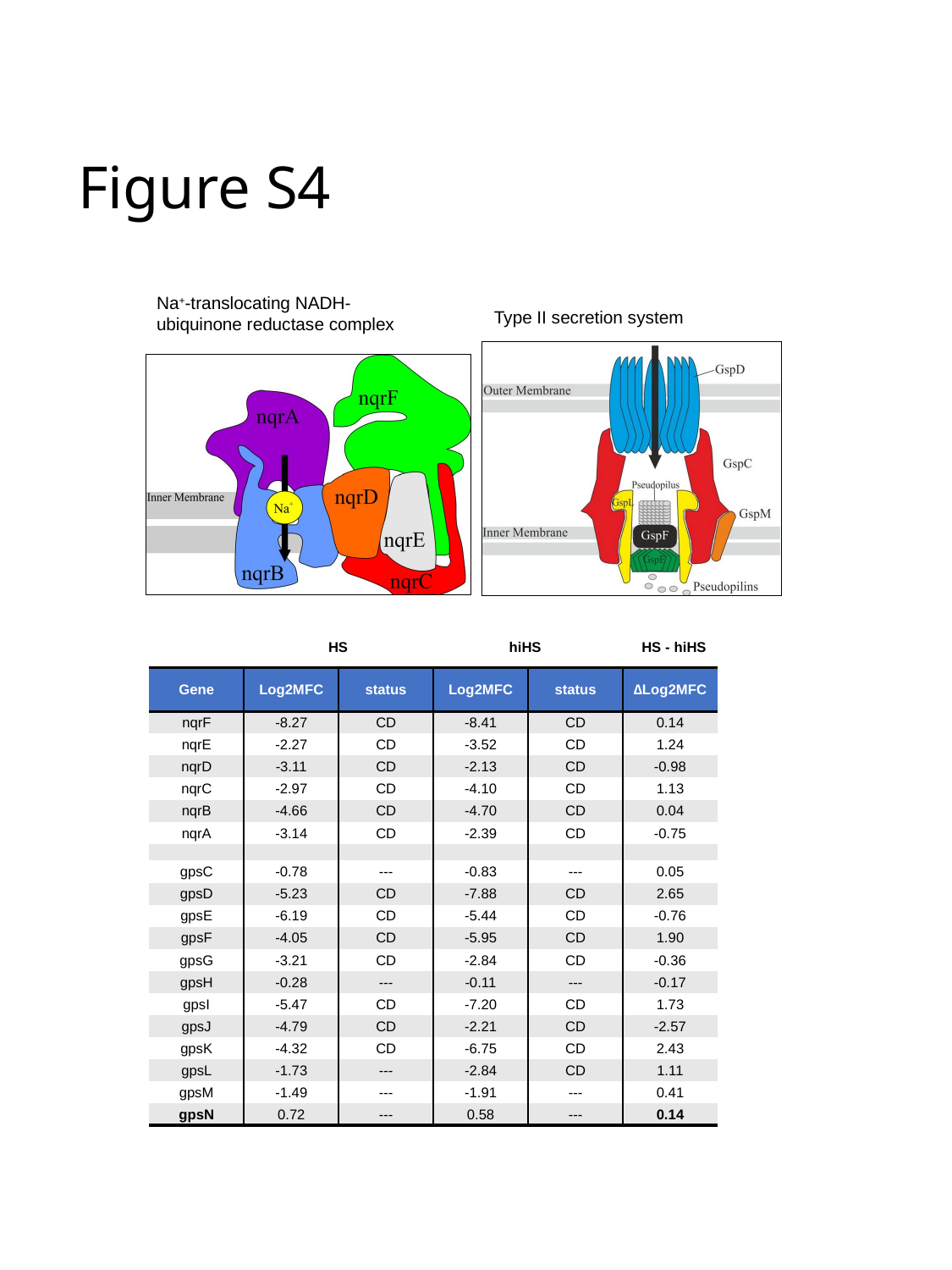

# Figure S4
Na+-translocating NADH-ubiquinone reductase complex
Type II secretion system
HS
hiHS
HS - hiHS
| Gene | Log2MFC | status | Log2MFC | status | ∆Log2MFC |
| --- | --- | --- | --- | --- | --- |
| nqrF | -8.27 | CD | -8.41 | CD | 0.14 |
| nqrE | -2.27 | CD | -3.52 | CD | 1.24 |
| nqrD | -3.11 | CD | -2.13 | CD | -0.98 |
| nqrC | -2.97 | CD | -4.10 | CD | 1.13 |
| nqrB | -4.66 | CD | -4.70 | CD | 0.04 |
| nqrA | -3.14 | CD | -2.39 | CD | -0.75 |
| | | | | | |
| gpsC | -0.78 | --- | -0.83 | --- | 0.05 |
| gpsD | -5.23 | CD | -7.88 | CD | 2.65 |
| gpsE | -6.19 | CD | -5.44 | CD | -0.76 |
| gpsF | -4.05 | CD | -5.95 | CD | 1.90 |
| gpsG | -3.21 | CD | -2.84 | CD | -0.36 |
| gpsH | -0.28 | --- | -0.11 | --- | -0.17 |
| gpsI | -5.47 | CD | -7.20 | CD | 1.73 |
| gpsJ | -4.79 | CD | -2.21 | CD | -2.57 |
| gpsK | -4.32 | CD | -6.75 | CD | 2.43 |
| gpsL | -1.73 | --- | -2.84 | CD | 1.11 |
| gpsM | -1.49 | --- | -1.91 | --- | 0.41 |
| gpsN | 0.72 | --- | 0.58 | --- | 0.14 |
